# Hyperactivation and *in situ* recruitment of inflammatory Vδ2 T cells contributes to disease pathogenesis in systemic lupus erythematosus

**DOI:** 10.1038/srep14432

**Published:** 2015-09-23

**Authors:** Shanshan Yin, Yujia Mao, Xuemei Li, Cai Yue, Chen Zhou, Linfang Huang, Wenxiu Mo, Di Liang, Jianmin Zhang, Wei He, Xuan Zhang

**Affiliations:** 1 Department of Rheumatology & Clinical Immunology, Peking Union Medical College Hospital, Chinese Academy of Medical Sciences and Peking Union Medical College, Beijing, 100730, China; 2 Department of Immunology, School of Basic Medicine, Peking Union Medical College, and Institute of Basic Medical Sciences, Chinese Academy of Medical Sciences, Beijing, 100005, China; 3 Department of Nephrology, Peking Union Medical College Hospital, Chinese Academy of Medical Sciences and Peking Union Medical College, Beijing, 100730, China

## Abstract

In this study, we measured the proportion of peripheral Vδ2 T cells as well as the status and chemokine receptor expression profiles in SLE patients and healthy control (HC). In addition, Vδ2 T cell infiltration in the kidneys of patients with lupus nephritis was examined. The results showed that the percentage of peripheral Vδ2 T cells in new-onset SLE was decreased, and negatively correlated with the SLE Disease Activity Index score and the severity of proteinuria. These cells had a decreased apoptosis but an increased proliferation, and they showed increased accumulation in SLE kidneys. Moreover, IL-21 production and CD40L, CCR4, CCR7, CCR8, CXCR1 and CX3CR1 expression in Vδ2 T cells from SLE patients was significantly higher than from HC (p < 0.05), and these factors were downregulated in association with the repopulation of peripheral Vδ2 T cells in patients who were in remission (p < 0.05). In addition, anti-TCR Vδ2 antibodies activation significantly upregulated these chemokine receptors on Vδ2 T cells from HC, and this effect was blocked by inhibitors of PLC-γ1, MAPK/Erk, and PI3K signaling pathways. Our findings demonstrate that the distribution and function status of Vδ2 T cells from SLE patients are abnormal, and these aberrations may contribute to disease pathogenesis.

According to the expression of rearranged adaptive T cell receptors (TCRs), T cells are divided into two major subsets, γδ T cells and αβ T cells^1^. γδ T cells, which represent a small subset (1%–10%) of CD3^+^ cells^2^, can be divided into two subsets: Vδ1 T cells, which primarily dwell in the epithelial-associated lymphoid tissue, and Vδ2 T cells, which are found in the peripheral blood^3^,^4^. Vδ1 T cells and Vδ2 T cells have considerable inherent differences in gene expression^5^; in addition, Vδ1 T cells and Vδ2 T cells recognize different TCR receptors. Most Vδ2 T cells are uniquely activated by P-Ags^6^, whereas Vδ1 T cells are thought to be activated by other microbial products^7^. These cell types also differ in cytokine production and receptor expression, as Vδ2 T cells are inflammatory and Vδ1 T cells are regulatory^5^. Vδ1 T cells have been demonstrated to express Foxp3, and their number is substantially decreased in peripheral blood from patients with new-onset systemic lupus erythematosus (SLE)^8^,^9^. Vδ2 T cells have predominantly been investigated in tumor immunosurveillance and the host defense against viral invasion^10^,^11^,^12^,^13^. The precise role of Vδ2 T cells in the pathogenesis of SLE remains elusive.

SLE is a systemic autoimmune disease characterized by autoantibody production and deposition in tissues and organs^14^,^15^. T cells play an important role in inducing B cell hyperactivity^16^,^17^. Activated T cells express CD40L^18^, and the engagement of CD40L with CD40 on B cells promotes immunoglobulin (Ig) secretion and isotype switching^19^. Activated T cells also secrete IL-21, which plays a major role in co-stimulating B cells^20^,^21^. Studies have also shown that Vδ2 T cells from both peripheral blood and secondary lymphoid tissues express costimulatory factors, including inducible costimulator (ICOS) and CD40L, and produce Th2-type cytokines such as IL-4 and IL-10^22^,^23^,^24^. Coculture with activated Vδ2 T cells resulted in a substantial increase in the B cell-mediated production of IgG, IgA, and IgM^25^, suggesting that Vδ2 T cells may participate in a B cell hyperactivity network in SLE.

The local recruitment of inflammatory cells is also involved in tissue injury in SLE^26^,^27^. The *in situ* expression levels of the Vδ2 TCR^+^ and Vγ9 TCR^+^ were found to be higher in the skin of SLE patients and positively correlated with disease activity^28^. As Vδ2 T cells are primarily found in the peripheral blood, we were interested in exploring whether Vδ2 T cells can induce B cell hyperactivity to produce autoantibodies and whether Vδ2 T cells can be recruited to local tissues to directly participate in tissue injury in SLE.

In this study, we found that the number of circulating Vδ2 T cells in the peripheral blood of new-onset SLE patients was significantly reduced and negatively correlated with the SLE Disease Activity Index (SLEDAI) score and the severity of proteinuria. However, this reduction in circulating Vδ2 T cells was not caused by either increased apoptosis or decreased proliferation. Rather, we found a substantial accumulation of Vδ2 T cells in the kidneys of SLE patients. In addition, the expression profile of chemokine receptors on Vδ2 T cells was examined, and we found that the CCR4, CCR7, CCR8, CXCR1, CX3CR1, and CD40 L expression levels as well as IL-21 secretion by Vδ2 T cells were significantly elevated in SLE patients and were downregulated after treatment with corticosteroids and immunosuppressants. A mechanistic study showed that upon activation by an anti-TCR Vδ2 antibody, the expression levels of CCR4, CCR7, CCR8, CXCR1 and CX3CR1 on Vδ2 T cells were significantly increased. Additionally, this effect was blocked by PLC-γ1, MAPK/Erk, and PI3K inhibitors. Collectively, these data suggested that Vδ2 T cells can overexpress CD40L and IL-21, induce B cell hyperactivity, migrate to local tissues and act as inflammatory cells to directly participate in tissue injury in SLE, leading to disease exacerbation.

## Results

### Peripheral Vδ2 T cells were decreased in new-onset SLE patients

**Figure 1:**
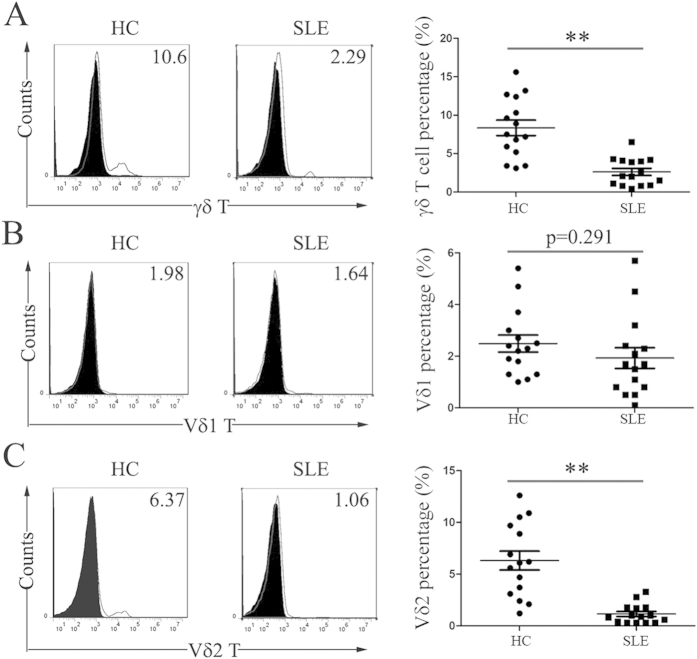
The percentage of peripheral Vδ2 T cells was decreased in new-onset SLE patients. Fresh PBMCs were stained with an anti-γδ TCR mAb, anti-Vδ1 mAb or anti-Vδ2 mAb and were analyzed with flow cytometry. The left panels show representative dot plots from flow cytometry for γδ T cells (**A**) Vδ1 T cells (**B**) or Vδ2 T cells (**C**). The right panels show bar graphs of the percentage of positively stained cells from 15 healthy adults (HC) and 15 SLE patients. **p < 0.01.

We first compared the percentages of total peripheral γδ T cells and the Vδ1 and Vδ2 T subsets between new-onset SLE patients and HC. Our results showed that there was a significant decrease in the percentage of total γδ T cells in the peripheral blood of new-onset SLE patients (2.61 ± 1.79% vs. HC 8.35 ± 3.91%, p<0.01) (Fig. 1A). This decrease was primarily due to a reduction in Vδ2 T cells (1.15±0.94% vs. HC 6.31 ± 3.55%, p < 0.01) but not in Vδ1 T cells (1.93 ± 1.55 vs. HC 2.49 ± 1.28, p = 0.291) (Fig. 1B,C). The absolute numbers of Vδ2 T cells in new-onset SLE patients was also lower than that in HC (134.3 ± 16.2 vs. HC 414.5 ± 57.3, p < 0.01).

### Decreased apoptosis and increased proliferation of Vδ2 T cells in new-onset SLE patients

**Figure 2:**
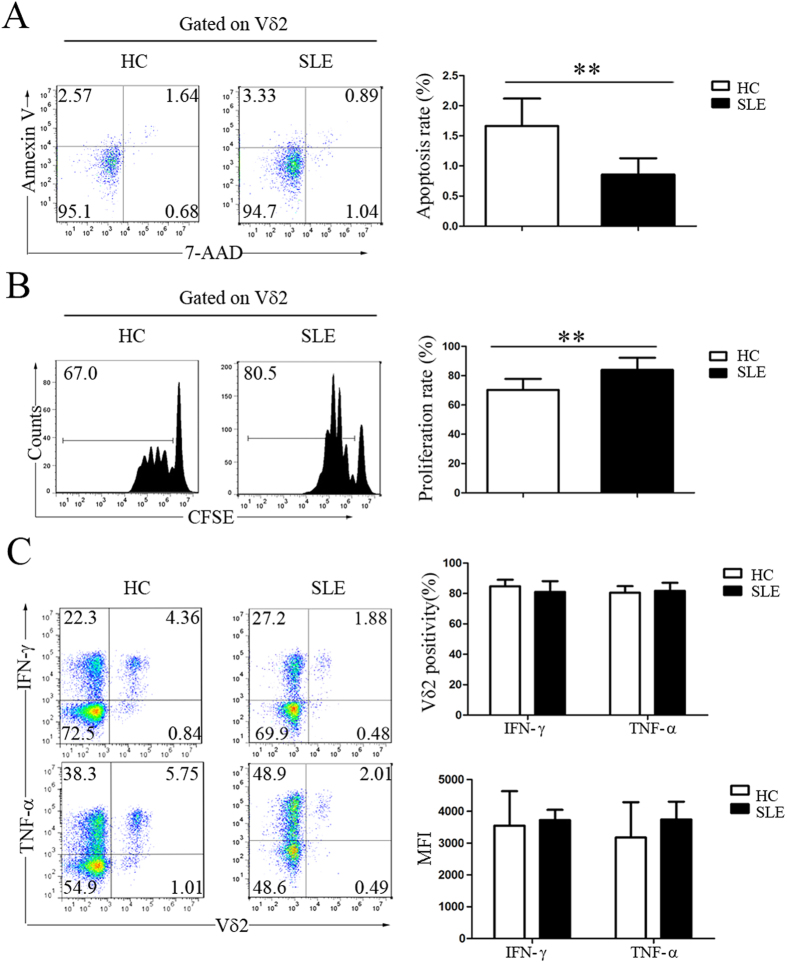
The decrease in peripheral Vδ2 T cells in SLE patients was not caused by either increased apoptosis or decreased proliferation. (**A**) Fresh PBMCs from healthy controls (HC) and SLE patients were stained with an anti-Vδ2 mAb, 7-AAD and Annexin V. The data were gated for Vδ2 T cells. The frequency of 7-AAD and Annexin V double-positive labeling represents apoptosis. (**B**) Fresh PBMCs were labeled with CFSE and expanded using an immobilized anti-pan-TCRγδ mAb. (**C**) Intracellular staining for IFN-γ and TNF-α in Vδ2 T cells. **p < 0.01.

To examine why peripheral Vδ2 T cells were reduced in SLE patients, we first measured the apoptosis and proliferation rates of these cells. By performing Annexin V/7–AAD double-staining flow cytometric analysis, we found that the apoptosis rate of peripheral Vδ2 T cells in new-onset SLE patients was not increased but rather was decreased compared with that in HC (0.86 ± 0.27% vs. 1.66 ± 0.46%, p < 0.01) (Fig. 2A). In addition, the proliferation rate of Vδ2 T cells in new-onset SLE patients was significantly increased compared with that in HC (83.97 ± 8.26% vs. 70.27 ± 7.55%, p < 0.01) (Fig. 2B). When we assessed the activation status of Vδ2 T cells, we found that most Vδ2 T cells secreted IFN-γ and TNF-α and that the Vδ2 T cell-mediated production of IFN-γ and TNF-α in SLE patients did not differ from that in HC (Fig. 2C). These data collectively suggested that the peripheral reduction in Vδ2 T cells was probably due to other causes, such as increased recruitment of these cells to local tissues.

### Percentage of peripheral Vδ2 T cells negatively correlated with disease activity and the severity of proteinuria

**Figure 3:**
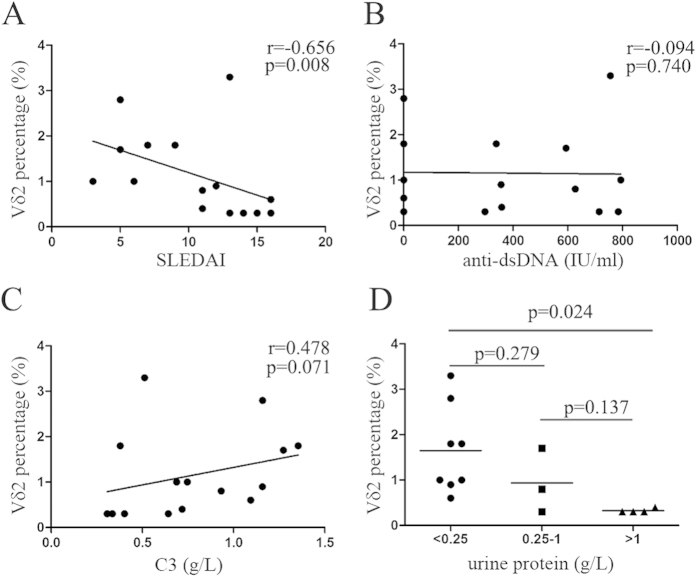
Percentage of peripheral Vδ2 T cells negatively correlated with disease activity and the severity of proteinuria.

The percentage of peripheral Vδ2 T cells in SLE patients negatively correlated with disease activity, as determined by the SLEDAI score (r = −0.656, p = 0.008, Fig. 3A), but not with the titer of anti-dsDNA autoantibodies or the serum C3 levels (p > 0.05, Fig. 3B,C). Notably, the percentage of Vδ2 T cells significantly correlated with the severity of nephritis, which manifested as proteinuria (p < 0.05, Fig. 3D).

### Increased CD40L expression and IL-21 production by Vδ2 T cells from SLE patients

**Figure 4:**
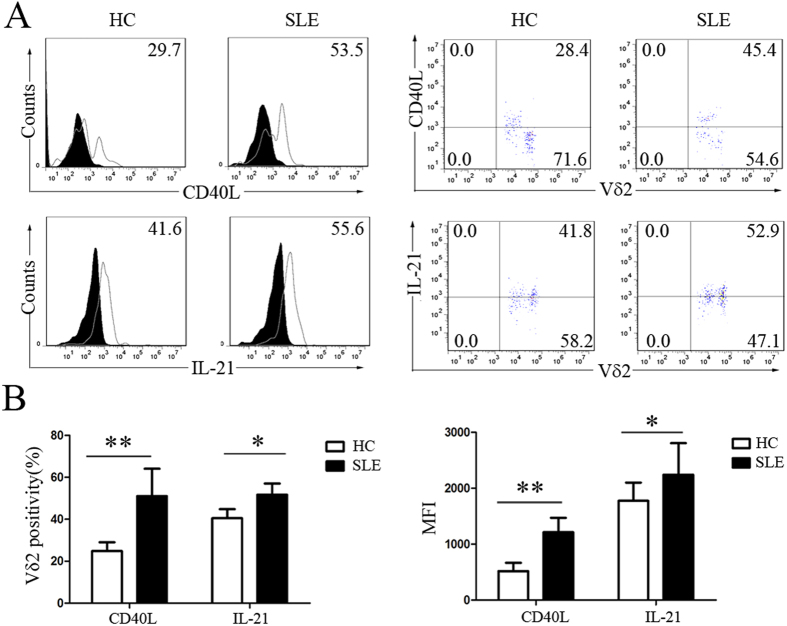
Increased CD40L expression and IL-21 production by Vδ2 T cells from SLE patients. (**A**) CD40L was primarily expressed on Vδ2^low^ T cells. Fresh Vδ2 T cells were stained with a mAb against Vδ2 and CD40L or IL-21. The left panel compares the expression of CD40L and IL-21 between healthy adults and SLE patients. The right panel compares the expression of CD40L or IL-21 between Vδ2^low^ T cells and Vδ2^high^ T cells. The data were gated for Vδ2 T cells. (**B**) The percentage and MFI of CD40L^+^/IL-21^+^ cells among the total Vδ2 T cells freshly isolated from HC and SLE patients. *p < 0.05, **p < 0.01.

To determine whether Vδ2 T cells can induce B cell hyperactivation by upregulating costimulatory factors, we examined the CD40L expression and IL-21 production abilities of Vδ2 T cells. Our results showed that CD40L was primarily expressed by Vδ2 T cell subsets that had low TCR Vδ2 expression and that the level of CD40L expression by Vδ2 T cells from SLE patients was significantly higher than from HC (51.05 ± 13.04% vs. 24.80 ± 4.25%, p < 0.01) (Fig. 4A,B). The percentage of Vδ2 T cells that were positive for intracellular IL-21 in SLE patients was significantly higher than in HC (51.69 ± 5.35% vs. 40. 52 ± 4.32%, p < 0.05) (Fig. 4A,B).

### Aberrant chemokine receptor expression profiles in Vδ2 T cells from SLE patients

**Figure 5:**
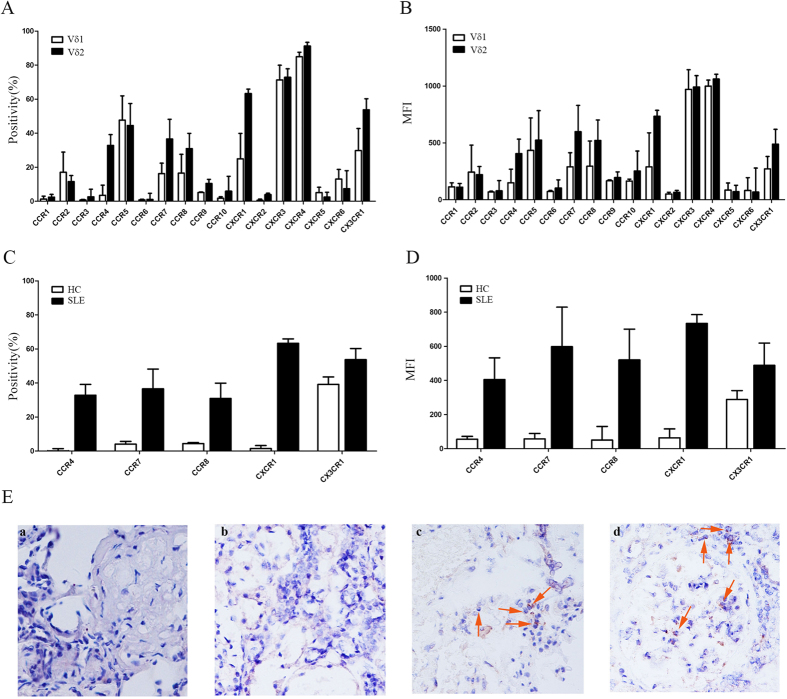
Vδ2 T cells from SLE patients had an aberrant chemokine receptor expression profiles and were increasingly recruited to the kidneys. (**A**,**B**) The percentage and MFI of Vδ1/Vδ2 T cells from SLE patients that were positive for individual chemokine receptors (n = 15); (**C**,**D**) Comparison of the positivity and MFI of individual chemokine receptor expression on Vδ2 T cells between SLE patients and HC; (**E**) Vδ2 T cells recruitment in the kidney of lupus nephritis (c,d) but not kidney of disease control (a: IgA nephrosis; b: allergic purpuric nephritis).

Chemokine receptors, including CCR1-10, CXCR1-6 and CX3CR1, play a central role in cell trafficking to local tissues. Thus, we investigated the chemokine receptor expression profile of Vδ2 T cells. Our results showed that in SLE patients, CCR4, CCR7, CCR8, CXCR1 and CX3CR1 expressed on Vδ2 T cells were significantly higher than Vδ1 T cells (p < 0.05) (Fig. 5A–D). In addition, the expression levels of CCR4, CCR7, CCR8, CXCR1 and CX3CR1 by Vδ2 T cells from SLE patients were significantly higher than those from HC (p < 0.05) (Fig. 5C,D), suggesting increased Vδ2 T cell trafficking to local tissues in SLE patients. This hypothesis was confirmed by an immunohistochemical assay demonstrating an increase in the accumulation of Vδ2 T cells in the kidneys of patients with lupus nephritis (Fig. 5E).

### Anti-TCR Vδ2 activating antibodies significantly upregulated chemokine receptor expression in Vδ2 T cells

**Figure 6:**
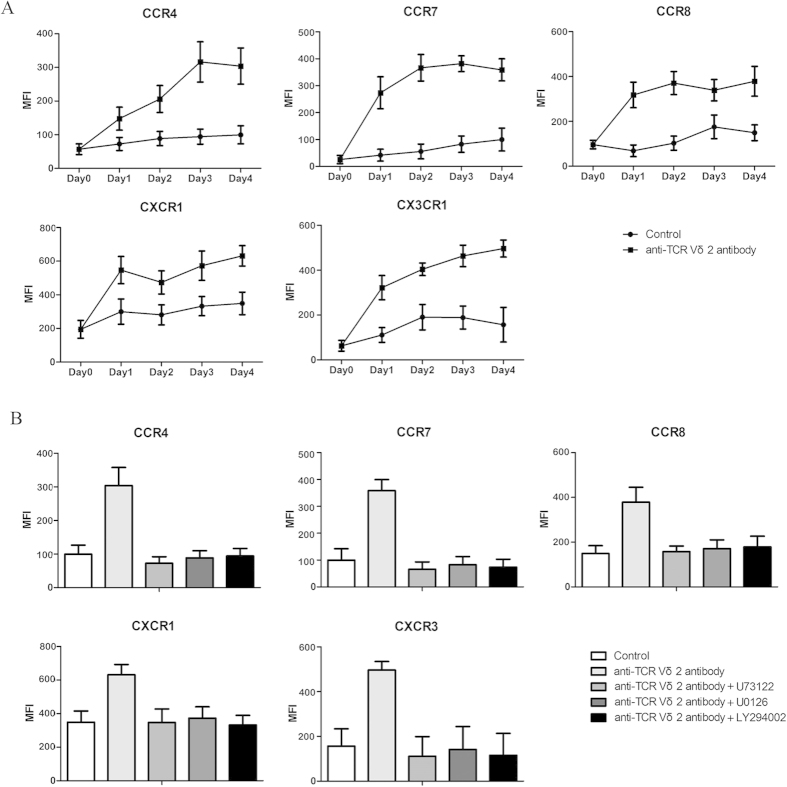
Anti-TCR Vδ2 activating antibodies significantly upregulated chemokine receptor expression in Vδ2 T cells. (**A**) Flow cytometric analysis of chemokine receptor expression on normal Vδ2 T cells stimulated with an anti-TCR Vδ2 antibody; (**B**) Flow cytometric analysis of chemokine receptor expression on normal Vδ2 T cells exposed to 5 μM U73122, 30 μM UO126, or 50 μM LY294002 before stimulation with an anti-TCR Vδ2 antibody.

To investigate whether activation of Vδ2 T cells could induce upregulation of chemokine receptors, we stimulated Vδ2 T cells with anti-TCR Vδ2 activating antibodies, and found that the expression levels of CCR4, CCR7, CCR8, CXCR1 and CX3CR1 on Vδ2 T cells were upregulated by immobilized anti-TCR Vδ2 antibodies but not IgG1 isotype control (Fig. 6A). Three important signal pathways, PLC-γ1, MAPK/Erk, and PI3K, are involved in T cell activation^29^. So we applied U73122 (PLC-γ1 inhibitor), UO126 (MAPK/Erk inhibitor), and LY294002 (PI3K inhibitor), and investigated their roles in the upregulation of chemokine receptors on activated Vδ2 T cells, and we found each signaling inhibitor completely abrogated the upregulation of chemokine receptors on Vδ2 T cells (Fig. 6B).

### Repopulation of peripheral Vδ2 T cells with downregulated levels of chemokine receptors and costimulatory factors in remission SLE patients after treatment

**Figure 7:**
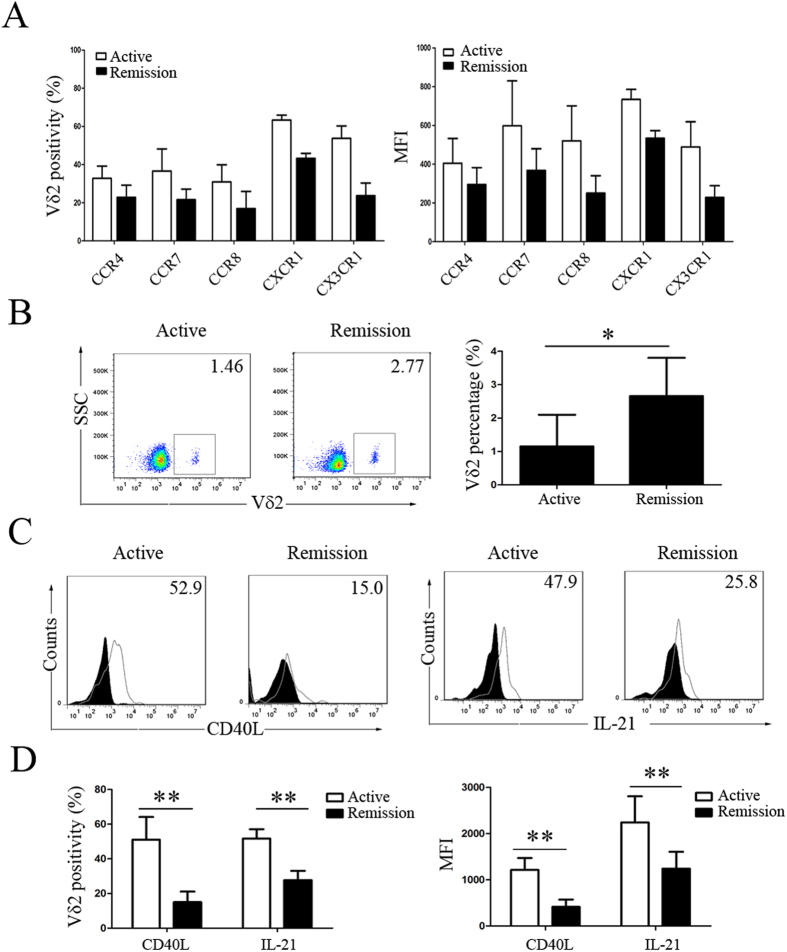
Repopulation of peripheral Vδ2 T cells with downregulated levels of chemokine receptors and costimulatory factors in SLE patients in remission. Comparison of (**A**) the positivity and MFI of individual chemokine receptor expression on Vδ2 T cells, (**B**) the percentage of peripheral Vδ2 T cells, and (**C**,**D**) the positivity and MFI of CD40L and IL-21 expression in Vδ2 T cells between new-onset active and remitted SLE patients. The filled graphs represent isotype controls, and the unfilled graphs represent CD40L or IL-21staining. *p < 0.05, **p < 0.01.

In SLE patients who were in remission after treatment with corticosteroids and immunosuppressant, the expression levels of CCR4, CCR7, CCR8, CXCR1 and CX3CR1 on Vδ2 T cells were downregulated (Fig. 7A), and the percentage of peripheral Vδ2 T cells was increased but did not return to normal (Figs 1C and 7B). Moreover, the Vδ2 T cell-mediated expression of CD40L and production of IL-21 were downregulated in SLE patients after treatment (15.06 ± 6.04% vs. 51.06 ± 13.04% and 27.69 ± 5.35% vs. 51.69 ± 13.04%, respectively, p < 0.05) (Fig. 7C–D).

## Discussion

Although there have been a few studies investigating γδ T cells in SLE patients, the results have been inconsistent^8^,^30^,^31^. One of the reasons may be that all of the previous studies examined total peripheral γδ T cells without dissecting Vδ1 and Vδ2 T cells, even though these two cell types are phenotypically and functionally distinct, as Vδ1 T cells are immunoregulatory and Vδ2 T cells are inflammatory^5^. By examining new-onset patients, our study demonstrated that the number of peripheral γδ T cells was significantly decreased due to a reduction in Vδ2 T cells but not in Vδ1 T cells. In addition, we found that the decrease in Vδ2 T cells was not caused by either increased apoptosis or impaired proliferation. Rather, the apoptosis rate was decreased and the proliferation rate was increased, suggesting an abnormal overgrowth of inflammatory Vδ2 T cells in SLE.

In this study, we found that the reduction in Vδ2 T cells correlated with the severity of proteinuria and that Vδ2 T cell infiltration into the kidneys was increased in patients with lupus nephritis, suggesting that Vδ2 T cells are abnormally recruited to tissues to participate in tissue damage in SLE patients. In fact, the recruitment of γδ T cells (including Vδ2 T cells) to local organs and tissues has been reported in some diseases^28^,^32^,^33^,^34^,^35^,^36^,^37^,^38^,^39^. Selective Vδ2 T cell homing from the blood to tissues is controlled by a multi-step process involving interactions between chemokines and chemokine receptors. Jiang *et al.* reported that the accumulation of γδ T cells in the embryo is CCR4-dependent^40^. During an allergic reaction, CCL25 drives the mobilization of IL-17^+^ γδ T cells to inflamed tissue^34^. Additionally, through CCL2-CCR2 interactions, γδ T cells can be recruited during allergic inflammation^41^. Our study demonstrated that Vδ2 T cells express a variety of chemokine receptors and that the expression levels of CCR4, CCR7, CCR8, CXCR1 and CX3CR1 on Vδ2 T cells are upregulated in new-onset SLE patients. Similar to the abnormal activation state of Vδ2 T cells in SLE patients, we found that antibody-induced T cell stimulation significantly upregulated the expression levels of CCR4, CCR7, CCR8, CXCR1 and CX3CR1 on Vδ2 T cells from HC. In SLE patients in remission after effective treatment with glucocorticoids and immunosuppressant, the overexpression of these chemokine receptors was rectified in association with the repopulation of peripheral Vδ2 T cells, further supporting the abnormal local trafficking of these inflammatory Vδ2 T cells in SLE patients.

The hyperactivation and dysfunction of B cells, which ultimately leads to the massive production of autoantibodies, is the major pathological event in SLE^42^. CD40L, IL-21, and other costimulatory factors such as ICOS play pivotal roles in controlling B cell homeostasis and function, and numerous reports have suggested that the abnormal regulation of CD40L, IL-21, and ICOS is implicated in B cell hyperactivation in SLE^22^,^42^,^43^. Previous studies showed that Vδ2 T cells from both peripheral blood and secondary lymphoid tissues express both ICOS and CD40L^22^ and produce Th2-type cytokines such as IL-4 and IL-10^23^,^24^. Our results showed that Vδ2 T cells from SLE patients overexpressed CD40L and produced increased levels of IL-21, which were downregulated during remission after treatment, suggesting that Vδ2 T cells are important in promoting B cell hyperactivation in SLE. In addition, via the upregulation of CCR4, CCR7, CCR8, CXCR1 and CX3CR1, these inflammatory Vδ2 T cells were abnormally recruited to local tissues, such as the kidneys, to participate in tissue damage and contribute to disease pathogenesis in SLE.

In summary, this study demonstrated that the percentage of peripheral Vδ2 T cells decreased significantly in new-onset active SLE patients. As these cells exhibited a decreased apoptosis rate, an increased proliferation rate, and upregulated levels of costimulatory factors including CD40L and IL-21, we suggest that these overgrown and hyperactive inflammatory Vδ2 T cells are increasingly recruited to tissues via the upregulation of a variety of chemokine receptors to participate in inflammation and tissue damage in SLE.

## Materials and Methods

### Patients and controls

This study was approved by the Institutional Review Board of Peking Union Medical College Hospital and written informed consent was obtained from each participating patient and healthy control (HC). And the methods were carried out in accordance with the approved guidelines. Fifteen patients with new-onset SLE (including 14 females and 1 male; mean age 24.8 y; range 14–40 y; Table 1) from Peking Union Medical College (PUMC) Hospital were enrolled in this study, and peripheral blood samples were collected from these patients before and after treatment with glucocorticoids and immunosuppressant. All patients fulfilled the revised American College of Rheumatology criteria for SLE. Lupus disease activity was evaluated using the SLEDAI score (before treatment: mean 10.4, range 3–16; after treatment: 0–3). Peripheral blood samples were also collected from 15 healthy subjects as controls (HC) (14 females and 1 male; mean age 27 y; range 19–38 y).

### Antibodies and reagents

RPMI-1640 medium and fetal bovine serum (FBS) were obtained from Gibco; the anti-TCR Vδ2 antibody (ab171103) was obtained from Abcam; FITC-conjugated anti-human TCRγδ (IMMU510) was purchased from Beckman Coulter Immunotech; APC-conjugated anti-human CD3 (HIT3a), FITC-conjugated anti-human TCR Vδ2 (B6), PE-conjugated anti-human TCR Vδ2 (B6), APC/Annexin V Apoptosis Detection Kit with 7-AAD, DyLight™ 649-conjugated goat anti-mouse IgG (Poly4053), Alexa Fluor^®^ 647-conjugated anti-human IFN-γ (4S.B3), Alexa Fluor^®^ 647-conjugated anti-human TNF-α (MAb11), Alexa Fluor^®^ 647-conjugated anti-human CD40L (24-31), Alexa Fluor^®^ 647-conjugated anti-human IL-21 (3A3-N2), APC-conjugated anti-human CCR1 (5F10B29), APC-conjugated anti-human CCR2 (K036C2), PE-conjugated anti-human CCR3 (5E8), APC-conjugated anti-human CCR4 (L291H4), APC-conjugated anti-human CCR5 (J418F1), PE-conjugated anti-human CCR6 (G034E3), APC-conjugated anti-human CCR7 (G043H7), PE-conjugated anti-human CCR8 (L263G8), APC-conjugated anti-human CCR9 (L053E8), PE-conjugated anti-human CCR10 (6588-5), APC-conjugated anti-human CXCR1 (8F1/CXCR1), PE-conjugated anti-human CXCR2 (5E8/CXCR2), APC-conjugated anti-human CXCR3 (G025H7), PE-conjugated anti-human CXCR4 (12G5), PE-conjugated anti-human CXCR5 (J252D4), PE-conjugated anti-human CXCR6 (K041E5), PE-conjugated anti-human CX3CR1 (2A9-1) and Cell Activation Cocktail (with Brefeldin A) were purchased from Biolegend; the purified anti-TCR Vδ2 antibody (15D) was obtained from Abcam; FITC-conjugated anti-human TCR Vδ1 (TS8.2) was obtained from Pierce; CellTrace™ CFSE Cell Proliferation Kit was purchased from Invitrogen; and U73122, UO126, LY294002, phorbol myristate acetate (PMA) and ionomycin (Ion) were obtained from Sigma.

### Cells

Human peripheral blood mononuclear cells (PBMCs) from SLE patients and HC were collected in sodium heparin tubes (BD) and were purified via Ficoll-Hypaque (TBD, Tianjin, China) density gradient centrifugation. For the activation assay, fresh Vδ2 T cells (>90%) were purified by positive selection using an anti-FITC microbeads (MiltenyiBiotec), and then cultured in RPMI 1640 medium containing 10% fetal calf serum FBS in 24-well culture plates coated with 2 μg/ml anti-TCR Vδ2 antibody. After 4 days in culture, the expression levels of chemokine receptors were analyzed via flow cytometric analysis.

### Flow cytometric analysis

PBMCs were washed with PBS containing 1% bovine serum albumin (BSA) and were incubated in various combinations of monoclonal antibodies (mAbs) for 30 min at 4 °C. Then, the cells were washed and suspended in PBS. The stained cells were immediately analyzed with a BD Accuri C6 flow cytometer (Becton Dickinson) or were fixed in 1% paraformaldehyde and analyzed within 24 h. Data analysis was performed using FlowJo Software (Tree Star Inc.). For the CD40L expression assay, cells were pretreated with PMA (0.5 mg/ml) and Ion (1 mg/ml) for 5 h.

### Apoptosis assay

First, 1.0 × 10^7^ cells were washed twice with cold PBS containing 1% BSA, and then suspended with 100 μl Annexin V Binding Buffer. Then, 5 μl of APC/Annexin V, 5 μl of 7-AAD Viability Staining Solution and 5 μl of FITC-conjugated anti-human TCR Vδ2 antibody were added. The cells were gently vortexed and incubated for 15 min at room temperature (25 °C) in the dark. A total of 400 μl of Annexin V Binding Buffer was added to each tube. The stained cells were immediately analyzed with a BD Accuri C6 flow cytometer (Becton Dickinson). Data analysis was performed using FlowJo Software (Tree Star Inc.).

### Immunohistochemistry assay

Immunohistochemical staining was performed at the Department of Pathology Center, Peking Union Medical College Hospital, Chinese Academy of Medical Sciences and Peking Union Medical College. To block endogenous peroxidase activity, the samples were treated with 1.5% H_2_O_2_ for 20 min. Then, the slides were washed with TBS 3 times for 3 minutes each. Non-specific binding sites were blocked with 10% diluted goat serum in TBS for 20 hours. The samples were incubated overnight at 4 °C in the appropriate antibody dilution (1:5). The slides were washed 5 times in TBS and incubated in the anti-mouse IgG peroxidase antibody for 1 hour. The slides were washed 5 times in TBS. Avidin-biotin-peroxidase reagents were added, and the resulting peroxidase activity was revealed by incubating the slides in a 0.5 mg/mL HRP substrate solution (DAB + H_2_O_2_ prepared in distilled water). The slides were washed 5 times in TBS and counterstained for 1 minute with hematoxylin. The slides were dehydrated by washing the slides in an ethanol series for 1 minute each in 75%, 80% and, finally, 100% ethanol.

### Flow cytometric analysis

For the detection of intracellular IFN-γ, TNF-α and IL-21 in Vδ2 T cells, the cells were pretreated with Cell Activation Cocktail (with Brefeldin A) for 6 h. Then, the cells were washed twice with PBS containing 1% BSA and stained for surface molecules of Vδ2. After washing twice with PBS containing 1% BSA, the cells were fixed and permeabilized with BD cytofix/cytoperm solution. The cells were then washed with permeabilization solution before staining with anti-human IFN-γ, TNF-α and IL-21 antibodies. The cells were incubated at room temperature (25 °C) in the dark for 30 min. The cells were washed twice with permeabilization solution before analysis using a BD Accuri C6 flow cytometer (Becton Dickinson). Data analysis was performed using FlowJo Software (Tree Star Inc.).

### CFSE proliferation assay

PBMCs were labeled with CFSE and used as the responder cells. The cells were cultured in RPMI-1640 medium containing 10% FBS and 200 IU/ml IL-2 in 24-well culture plates coated with 1 μg/ml anti-pan-TCRγδ mAb in the dark. After 5 days in culture, the cells were collected, washed twice with PBS containing 1% BSA, and stained with a primary antibody against Vδ2 for 1 h at 4 °C. Then, the cells were stained with DyLight™ 649-conjugated goat anti-mouse IgG for 30 min at 4 °C following a thorough washing. The cells were analyzed using a BD Accuri C6 flow cytometer (Becton Dickinson) with gating of the Vδ2-positive cells. Data analysis was performed using FlowJo Software (Tree Star Inc.).

### Statistical analysis

All data were analyzed using SPSS 16.0 software. The results are expressed as the means ± SD, and one-way analyses of variance were used to compare data displaying a normal distribution and homogeneity of variance. Independent-sample t-tests were used to compare differences between two groups and differences before and after treatment. Correlations were calculated using Pearson’s correlation analysis. In all analyses, the minimum acceptable level of significance was p < 0.05.

## Additional Information

**How to cite this article**: Yin, S. *et al.* Hyperactivation and *in situ* recruitment of inflammatory Vd2 T cells contributes to disease pathogenesis in systemic lupus erythematosus. *Sci. Rep.* **5**, 14432; doi: 10.1038/srep14432 (2015).

## Figures and Tables

**Table 1:** Clinical profiles of new-onset SLE patients.

Patient No.	Gender/Age (y)	SLEDAI	Clinical manifestations	Therapy
SLE01	F/19	13.0	anti-dsDNA(+), C3↓, arthritis, hematuria, fever	Pred+HCQ+CTX
SLE02	F/24	9.0	C3↓, hematuria, alopecia, fever	Pred+MMF+HCQ
SLE03	F/21	5.0	anti-dsDNA(+), alopecia, thrombocytopenia	Pred+HCQ
SLE04	F/33	13.0	anti-dsDNA(+), C3↓, arthritis, proteinuria, fever	Pred+LEF+HCQ
SLE05	F/20	11.0	anti-dsDNA(+), C3↓, proteinuria, alopecia, leucopenia	Pred+MMF
SLE06	M/27	15	anti-dsDNA(+), C3↓, arthritis, proteinuria, rash, fever	Pred+HCQ+CTX
SLE07	F/25	3.0	rash, fever	Pred+HCQ
SLE08	F/18	12.0	anti-dsDNA(+), arthritis, hematuria, alopecia	Pred+MMF+HCQ
SLE09	F/20	16.0	anti-dsDNA(+), C3↓, arthritis, hematuria, rash, fever, leucopenia	Pred+HCQ+CTX
SLE10	F/25	16.0	anti-dsDNA(+), C3↓, arthritis, proteinuria, rash, alopecia	Pred+LEF+HCQ
SLE11	F/40	6.0	arthritis, rash	Pred+CTX
SLE12	F/30	7.0	anti-dsDNA(+), C3↓, rash, leucopenia	Pred+HCQ
SLE13	F/37	14.0	anti-dsDNA(+), proteinuria, pyuria, alopecia, fever, leucopenia	Pred+MMF
SLE14	M/14	11.0	arthritis, proteinuria, rash, fever	Pred+MMF
SLE15	F/19	5.0	C3↓, rash, fever	Pred+HCQ

Pred: prednisone HCQ: hydroxychloroquine CTX: cyclophosphamide MMF: mycophenolate LEF: leflunomide.
